# Study on CFRP-Strengthened Welded Steel Plates with Inclined Welds Considering Welding Residual Stress

**DOI:** 10.3390/ma17081804

**Published:** 2024-04-14

**Authors:** Xinyu Ding, Xu Liang, Man-Tai Chen, Lili Hu

**Affiliations:** 1 State Key Laboratory of Ocean Engineering, Shanghai Jiao Tong University, Shanghai 200240, China; dingxinyu@sjtu.edu.cn (X.D.); liang-xu@sjtu.edu.cn (X.L.); mantai.chen@sjtu.edu.cn (M.-T.C.); 2 Laboratory for Digital Maintenance of Buildings and Infrastructure, Shanghai Jiao Tong University, Shanghai 200240, China

**Keywords:** welded steel plate, inclined weld, welding residual stress, FRP strengthening, tensile behavior

## Abstract

Welded steel plates are widely used in various structural applications, and the presence of inclined welds is often encountered in practical scenarios. Carbon fiber reinforced polymer (CFRP) has been proven to be effective for strengthening steel structures. However, the behavior of CFRP-strengthened welded steel plates with inclined welds, particularly considering the influence of welding residual stress, is limited. This paper aims to investigate the tensile behavior of CFRP-strengthened welded Q355 steel plates with inclined welds considering welding residual stress (WRS). First, WRS data were obtained by the X-ray diffraction (XRD) method at different locations. The maximum tensile and compressive residual stresses are 0.39 and 0.14 times the yield strength of the steel, respectively. Then, finite element models were established to investigate the effects of weld angles, weld width, and height on the WRS distribution of welded steel plates. Finally, the tensile performance of CFRP-strengthened welded plates with WRS was studied by numerical simulation. The results showed that the weld angles have little effect on the distribution pattern of residual stress but significantly affect the peak tensile WRS. When the weld angle changes from 0° to 60°, the peak tensile WRS decreases significantly from 0.32 to 0.06 times the yield strength of steel; furthermore, the influence of weld width and height on WRS is relatively limited. Under tension loading, the maximum stress occurs near the weld. The ends of the weld enter the yielding state later than the middle part of the weld due to the distribution of the WRS. As the weld angle increases and the length of the weld increases, the stress in the weld zone decreases, while the stress in the base material zone correspondingly increases. In addition, CFRP strengthening can reduce the magnitude of stress. This study provides preliminary references for understanding the tensile behavior of CFRP-strengthened welded steel plates with inclined welds.

## 1. Introduction

Steel structures need strengthening due to increased loads, aging and deterioration, seismic and wind consideration, and fatigue damage. Fiber-reinforced polymer (FRP) is manufactured using different polymer matrices and incorporated filling materials [1,2], which has been proven to be effective to strengthen steel structures [3,4,5,6]. For example, Amraei [7] studied the tensile behavior of carbon fiber reinforced polymer (CFRP)-strengthened high-strength steel plates through experiments, demonstrating a significant strengthening effect of CFRP on such components, with an increase in tensile stiffness of 53–55% and an ultimate tensile loading capacity of 28–42%. Tong [8] conducted fatigue tests on CFRP-strengthened butt-welded steel plates and found that triple-layered double-sided CFRP strengthening increased fatigue life by 34%. Zheng [9] studied the tensile behaviors of CFRP-strengthened steel plates and proposed a theoretical calculation method for stress-strain curves. Hosseini [10] studied FRP-strengthened welded steel tubular joints and proposed a calculation method for the stress concentration factor. These studies collectively highlight the significant benefits of FRP reinforcement in enhancing the mechanical properties of steel structures. However, to the best of the authors’ knowledge, there is currently no research on FRP-strengthened inclined welded steel plates.

Due to the uncertainty in the direction of applied forces, obliquely loaded welded joints are common in engineering, such as spiral welded steel tubes under axial tension or bending loads. However, there is a limited amount of research specifically addressing the effect of weld angle and welding residual stress (WRS) on the tensile behavior of FRP-strengthened welded steel plates. In this paper, these types of welds are referred to as “inclined welds” to differentiate them from directly loaded welded joints that bear loads perpendicular to the weld. Weld angle and WRS can have a significant impact on the performance of welded steel structures. The relevant mechanical studies on inclined butt-welded steel plates indicate that there are significant differences in the static and fatigue behavior between inclined welds and straight welds. In terms of tensile behavior, inclined welds exhibit higher strength compared to straight butt welds [11]. Additionally, the failure mode of inclined welds differs from that of straight butt welds: the fracture path of inclined welds is influenced by the weld angle. When the weld angle is of less than 60°, the fracture direction aligns with the weld direction. However, when the weld angle reaches 60°, the fracture path forms an oblique angle with the weld [12]. Additionally, tensile WRS can lower fatigue strength by increasing the average stress, while compressive WRS can decrease the stability of the ultimate load-carrying capacity. WRS can also cause initial defects such as cracking and deformation in structures. During the mechanical processing, the release of WRS can result in additional deviations [13].

This study aims to bridge the existing knowledge gap by conducting a comprehensive investigation on the tensile behavior of carbon-fiber-reinforced polymer (CFRP)-strengthened welded plates by considering the influence of weld angel and WRS. The X-ray diffraction method is used to obtain the WRS of the welded plate. Then, finite element analysis (FEA) is employed to simulate the realistic welding process, and the resulting stress distribution is mapped onto the tensile behavior analysis model. The mechanical performance of the CFRP-strengthened welded steel plates is assessed under tensile loading conditions. This study develops a new finite element model for simulating WRS in inclined welded steel plates, which is reliability-validated by experimental data. Additionally, this study provides new and profound insights into the influence of weld angle on WRS and how both weld angle and WRS affect CFRP-strengthened inclined welded steel plate. The above represents the innovative aspects of this paper.

## 2. Distribution of Welding Residual Stress

### 2.1. Experiment

#### 2.1.1. Materials and Methods

This experiment utilized Q355 steel, a commonly used material in structural engineering due to its robust mechanical properties. A standard tensile test was conducted [14]. The elastic modulus *E* and yield strength *f*_y_ of steel were 206 GPa and 383 MPa, respectively. Semi-automatic CO_2_ gas shielded welding was used, which is capable of accommodating complex or irregular-shaped welds. The welding parameters, including voltage and current, were set as 28 V and 260 A, respectively, and the welding wire energy input was 4.5 kJ/mm. The flow rate of the CO_2_ shielding gas was controlled within the range of 15–20 L/min. In the welding process, parameters such as welding current, voltage and wire feed speed are machine-controlled, which includes sensors and control systems to monitor and adjust the current and voltage to keep them as close as possible to the preset values Welding speed is controlled by welder. JQ-MG50-6 wire with a diameter of 1.2 mm was used as the welding wire. For assessing the WRS in the welded joints, an iXRD portable X-ray stress analyzer (Proto, New Britain, CT, USA) was employed. This advanced equipment is able to conduct non-destructive stress analysis, allowing for an accurate evaluation of the welded specimens without compromising their structural integrity.

#### 2.1.2. Specimen Design and Preparation

**Figure 1:**
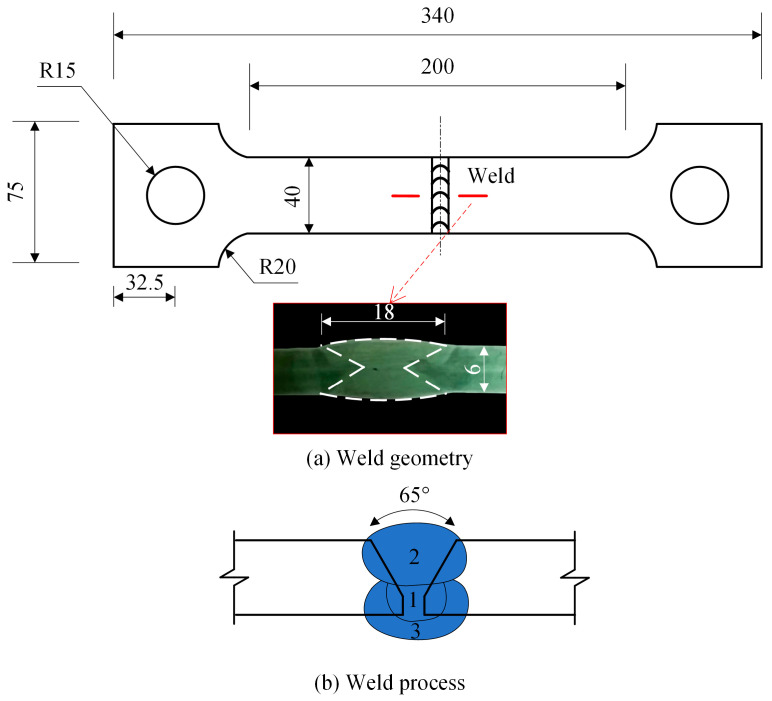
Butt-welded specimen (unit: mm).

This experiment focused on the straight butt weld specimen with dimensions referenced from previous studies [15,16] (Figure 1). The specimen was clamped at both ends, with the welding area situated in the middle. A Y-shaped joint configuration was designed, consisting of three layers and three steps as shown by the numbers 1, 2 and 3 in Figure 1b. The first step involved the root face on the front side, followed by the front filling. To ensure complete fusion of the weld, the third step was performed in the opposite direction after back-gouging the specimen.

#### 2.1.3. WRS Measurement and Results

X-ray diffraction (XRD) was employed in this study for measuring WRS [17]. XRD has the advantages of well-established principles, methodological advancements, repeatability, high precision, and a non-destructive nature. The principle of XRD is as follows: the wavelength of X-rays is comparable to the lattice spacing in the metal structure. When X-rays pass through the lattice, diffraction occurs, resulting in diffraction peaks. As strain is related to lattice spacing, changes in strain cause variations in lattice spacing, which consequently alter the position of the diffraction peaks. Therefore, by measuring the positions of the diffraction peaks, strain and stress can be determined.

**Figure 2:**
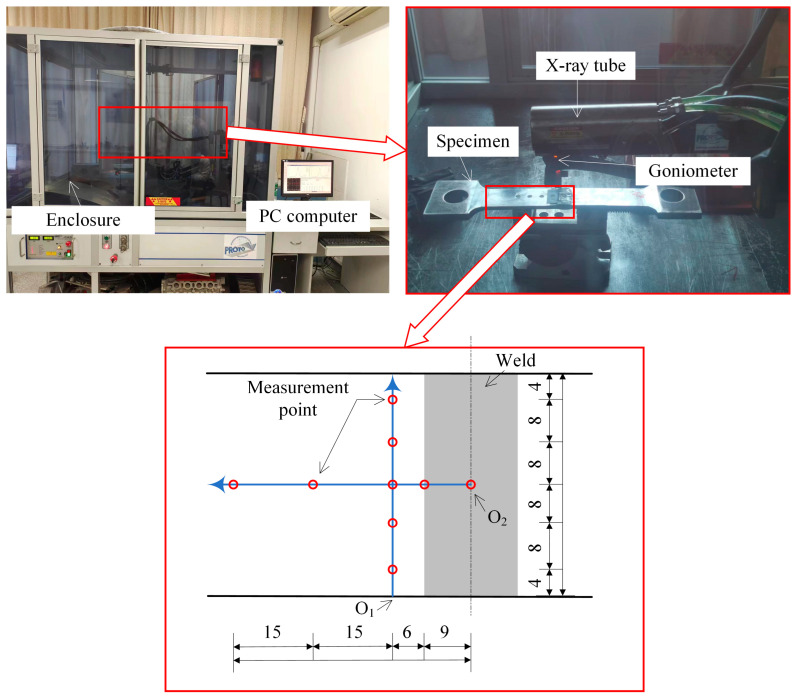
Measurement of WRS (unit: mm).

The X-ray stress measurement device consists of four components: an X-ray tube, a goniometer, an external PC, and a laboratory-grade enclosure, as shown in Figure 2. The measurement procedure is as follows: the specimen is placed on the platform inside the enclosure, the position of the X-ray tube is adjusted, and X-rays are emitted (the X-ray diameter spot on the material is approximately 3 mm). The goniometer measures the corresponding angles of the reflected peaks, and the PROTO XRDWIN software (https://www.protoxrd.com/products/xrd-software, accessed on 9 April 2024) installed on the PC automatically reads the data and calculates the WRS values. A total of nine measurement points were set to obtain the distribution of WRS in the weld and heat-affected zone. By connecting these nine points, two paths can be formed: one along the direction of the weld and the other perpendicular to the weld. O_1_ and O_2_ represent the starting points of these two paths, respectively. Due to the inconsistent geometric shape and poor flatness of the weld, the measured WRS can have significant errors. To improve the reliability of the data, the measurement points along the weld direction are shifted a short distance into the heat-affected zone. To eliminate the measurement error, measures such as etching and taking two measurements at the same point were adopted. Etching can help to remove surface contaminants and eliminate the impact of surface possible inhomogeneities on the results.

**Figure 3:**
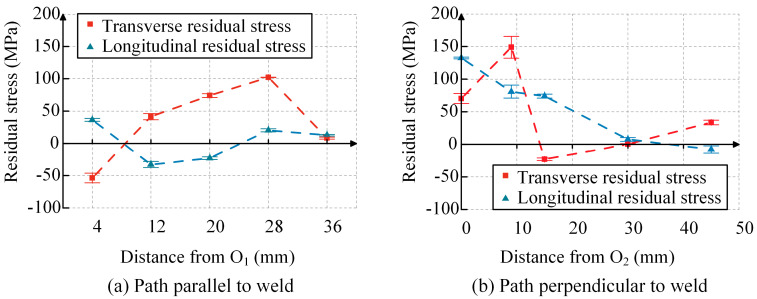
Results of WRS measurement.

A total of nine points representing WRS were obtained in two directions. Due to the continuity of stress distribution, the points along the two paths were connected to obtain the WRS distribution of the specimen, as shown in Figure 3. The longitudinal residual stress is defined as the WRS parallel to the weld direction, while the transverse residual stress is defined as the WRS perpendicular to the weld direction. Each data point represents the average of two measurement results with the error bar. From Figure 3a, it can be observed that along the path parallel to the weld, the transverse residual stress distribution at each point shows tensile stress at the center, compressive stress near the left end, and smaller tensile stress near the right end with a tendency towards compressive stress. The maximum tensile stress is 0.26*f*_y_, and the maximum compressive stress is 0.14*f*_y_. The longitudinal residual stress distribution at each point shows compressive stress at the center and tensile stress at the ends, with a maximum tensile stress of 0.09*f*_y_ and a maximum compressive stress of 0.08*f*_y_. From Figure 3b, it can be observed that along the path perpendicular to the weld, the transverse residual stress distribution at each point shows tensile stress at the center of the weld, reaching the maximum tensile stress at the weld toe. Then, it gradually transitions from tensile stress to compressive stress as it moves away from the center of the weld, and after reaching the maximum compressive stress, it tends towards zero and then transitions to smaller tensile stress. The maximum tensile stress is 0.39*f*_y_, and the maximum compressive stress is 0.06*f*_y_. The longitudinal residual stress distribution shows that the center of the weld experiences the maximum tensile stress, while the WRS away from the center of the weld are compressive. The maximum tensile stress is 0.34*f*_y_, and the maximum compressive stress is 0.02*f*_y_. These WRS distribution patterns align with the findings from previous studies [18,19].

### 2.2. Finite Element Analysis

#### 2.2.1. Model Details and Validation

**Figure 4:**
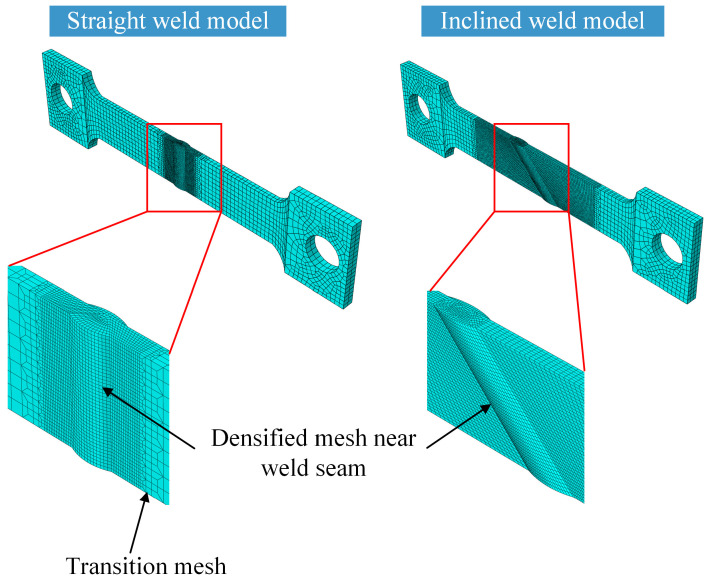
Mesh size.

The finite element simulation of WRS was conducted by Abaqus (Version of 6.8) [20]. The geometric dimensions and material parameters were set according to the actual specimen conditions. The simulation process of WRS involves the coupling of the temperature field and stress-strain field, which can be calculated by fully coupled or sequentially coupled methods. Due to its advantages of good convergence and high computational accuracy [18,19], this study adopts the sequentially coupled method for numerical simulation. In the sequentially coupled method, the temperature field is first calculated and imported into the model, followed by the calculation of the stress field. Therefore, the finite element model consists of a heat transfer model and a stress analysis model. The mesh sizes for both models are the same, as shown in Figure 4. The global mesh size for different weld angles is in the range of 2–4 mm, and the mesh is locally refined to around 1 mm in the weld area, ensuring mesh convergence. The heat transfer model uses 8-node hexahedral heat transfer elements (DC3D8), while the stress analysis model uses 8-node reduced integration 3D solid elements (C3D8R). The filling process of the weld is simulated using the birth and death element technique. All weld elements are killed before the calculation, and the killed weld elements are sequentially activated during the calculation. To address the issue of mesh distortion when activating the weld elements, the element birth and death technique is combined with the element erosion technique [21]. This method effectively solves the problem of mesh distortion caused by large deformations without affecting the original stress calculation results.

The transient temperature field variable *T* (*x*, *y*, *z*, *t*) in the three-dimensional problem of welding heat conduction analysis satisfies the following differential Equation (1) in the Cartesian coordinate system:

(1) $$\rho C\frac{\partial T}{\partial t}=\frac{\partial }{\partial x}(k\frac{\partial T}{\partial x})+\frac{\partial }{\partial y}(k\frac{\partial T}{\partial y})+\frac{\partial }{\partial z}(k\frac{\partial T}{\partial z})+Q$$

In the equation, *ρ*, *C*, and *k* represent the density, specific heat, and thermal conductivity of materials, respectively. *Q* represents the internal heat source intensity. The boundary conditions for this equation can be classified into three types: (1) the temperature on the boundary is known, (2) the heat flux density on the boundary is known, and (3) the convective heat transfer on the boundary is known. They can be expressed using Equations (2) and (3):

(2) $$T(x,y,z,0)=T_{0}$$

(3) $$k\frac{\partial T}{\partial x}N_{x}+k\frac{\partial T}{\partial y}N_{y}+k\frac{\partial T}{\partial z}N_{z}+q_{s}+h_{c}(T-T_{a})+\varepsilon_{\mathrm{em}}\sigma_{\mathrm{bol}}(T^{4}-T_{a}^{4})=0$$

In the equations, *T*_0_ represents the initial temperature field on the boundary; *N*_x_, *N*_y_, *N*_z_ are the direction cosines of the outward normal vector on the boundary; *q*_s_ is the heat flux density on the boundary; *h*_c_ is the convective heat transfer coefficient between the boundary surface and the surrounding air; *ε*_em_ is the effective thermal emissivity of the boundary surface; and *σ*_bol_ is the Boltzmann constant, with a value of 5.67 × 10^−11^ mW·mm^−2^·K^−4^.

**Figure 5:**
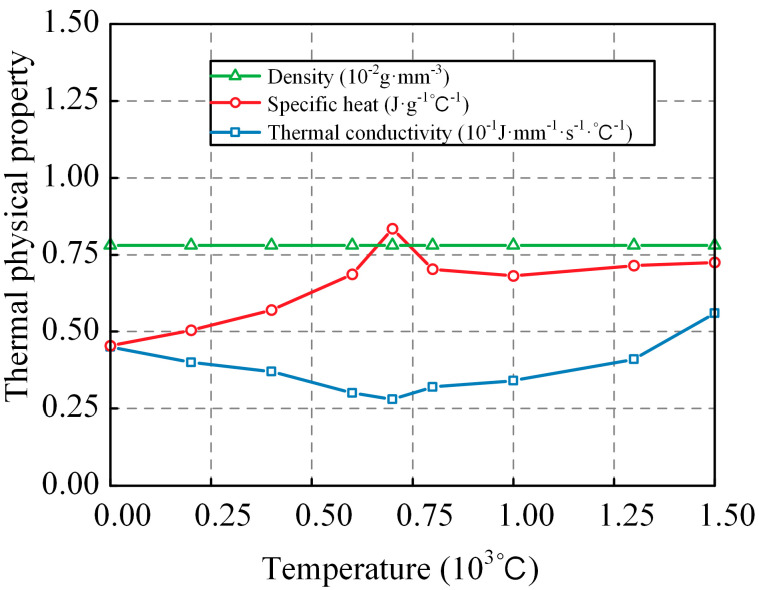
Thermal properties of steel and weld.

To reduce model complexity and decrease computation time, we assume that the thermal conductivity of the weld and base metal is identical and isotropic. When simulating the temperature field during the welding process, the values of the material density, specific heat, and thermal conductivity parameters [22] can be taken as shown in Figure 5. The initial ambient temperature of the specimen is 20 °C. The convective heat transfer coefficient between the steel material and air can be taken as 0.02 kW·mm^−2^·°C^−1^, and the effective thermal emissivity can be taken as 0.8 [23].

The heat source models used in welding include point heat source models, surface heat source models, and volumetric heat source models, among others. In simulations, the widely used double ellipsoid heat source model [24] can be applied. Equation (4) represents the expression for the heat flux density *q* in this model.

(4) $$q=\left\{\begin{array}{l} \frac{6\sqrt{3}(f_{f}nUI)}{a_{f}bc\sqrt{\pi }}\exp(-\frac{3x^{2}}{a_{f}^{2}}-\frac{3y^{2}}{{b_{h}}^{2}}-\frac{3z^{2}}{c^{2}})x\geq 0\\ \frac{6\sqrt{3}(f_{r}nUI)}{a_{r}bc\sqrt{\pi }}\exp(-\frac{3x^{2}}{a_{r}^{2}}-\frac{3y^{2}}{{b_{h}}^{2}}-\frac{3z^{2}}{c^{2}})x<0 \end{array}\right.$$

In the equation, *U* represents the welding voltage; *I* represents the welding current; *n* represents the heat efficiency; *f_f_* and *f_r_* represent the front and rear ellipsoid heat source distribution ratios, which can be taken as 0.6 and 1.4 [19]; and *a_f_*, *a_r_*, *b_h_*, and *c* represent the half-length, half-width, half-depth of the double ellipsoid. For this specific case, *b_h_* is taken as 8 mm, and *c* is taken as 4.5 mm. The values of *a_f_* = 8 mm and *a_r_* = 16 mm can be calculated using Equation (5).

(5) $$a_{f}=b_{h}=0.5a_{r}$$

The analysis of welding thermo-elastoplastic stresses mainly considers the thermal deformation and thermal stresses generated by the interaction of non-uniform temperature fields within the structure and external constraints. The constitutive equation [25] for this analysis is given by Equation (6):

(6) $$\varepsilon_{ij}=\frac{1}{2G}(\sigma_{ij}-\frac{v}{1+v}\sigma_{kk}\delta_{ij})+\alpha_{t}\Delta T\delta_{ij}+\varepsilon_{ij}^{p}$$

In the equation, *ε_ij_* and *σ_ij_* represent the strain and stress tensors, respectively, where *i*, *j* = 1, 2, 3; *σ_kk_* represents the sum of the principal stresses; *G* represents the shear modulus; *v* represents the Poisson’s ratio; *α*_t_ represents the coefficient of thermal expansion; Δ*T* represents the temperature change; *δ_ij_* represents the Kronecker delta symbol, which takes a value of 1 when *i* is equal to *j* and 0 otherwise; and $\varepsilon_{ij}^{p}$ represents the plastic strain.

**Figure 6:**
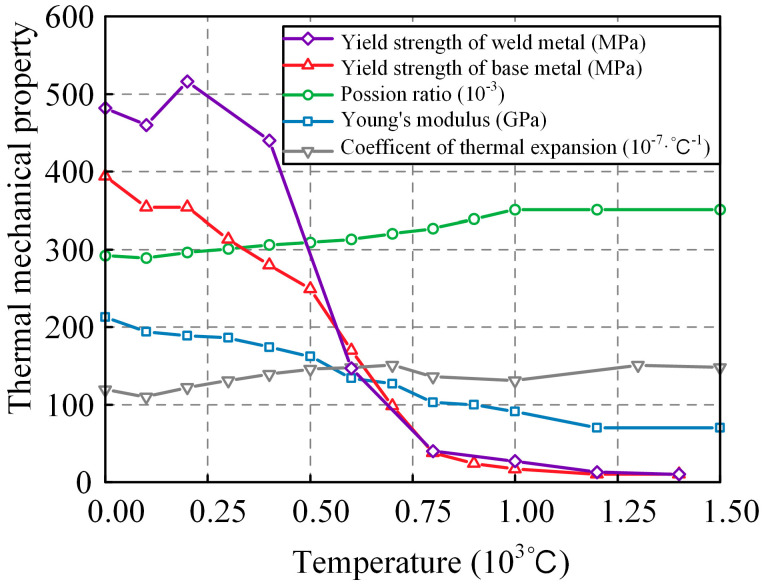
Mechanical properties of base metal and weld metal.

To solve for the WRS generated by welding, the temperature field results obtained from thermal conduction analysis are introduced into the thermal stress analysis model. The Von Mises yield criterion is used, and Figure 6 shows the mechanical parameters of the base metal and weld metal as a function of temperature [26]. The yield strength differs for the base metal and the weld metal.

**Figure 7:**
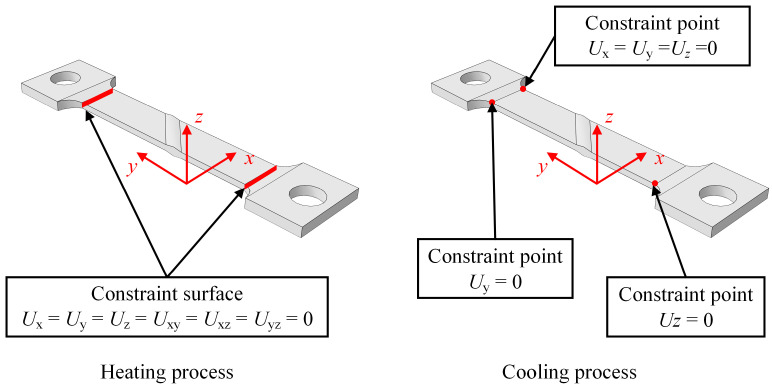
Boundary conditions for the thermal stress analysis.

The boundary conditions during the welding and cooling processes are completely fixed at both ends of the steel plate and rigid body displacement constraints are applied to the entire plate, respectively, as shown in Figure 7.

**Figure 8:**
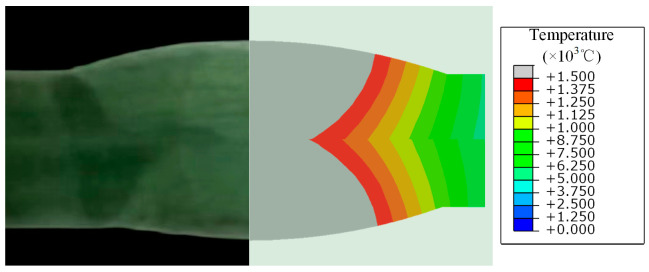
Comparison of melt pool shape between simulation and test results.

**Figure 9:**
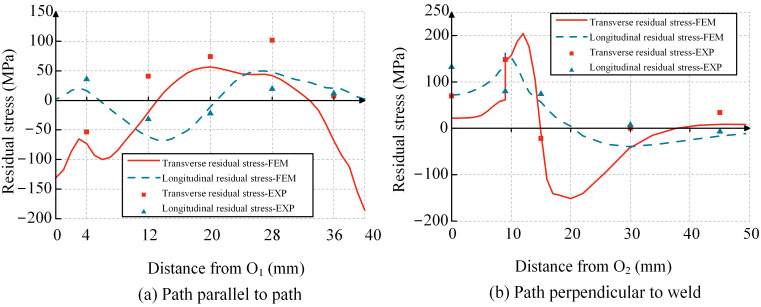
Comparison of WRS between simulation and test results.

**Figure 10:**
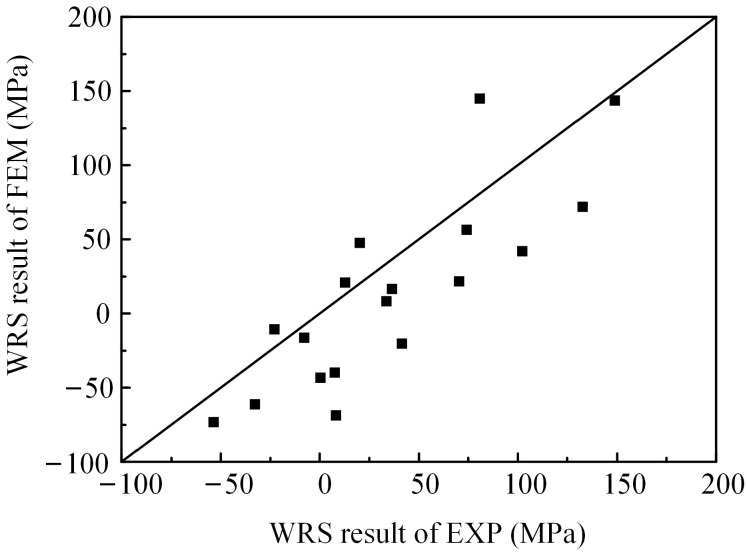
WRS results of FEM and EXP.

Using the straight weld model for verification against experimental results, Figure 8 compares the simulated and actual weld pool shapes, showing a good agreement between the simulation and the real component. The comparison of finite element and experimental WRS results along the corresponding path is shown in Figure 9, where FEM and EXP represent the finite element method and experimental results, respectively. It can be observed that the FEM results are close to theEXP results. Based on Equation (7), we calculated the mean absolute error (MAE) as 33.92 MPa. Figure 10 also shows the degree of deviation between the FEM results andEXP results at the measurement points. In addition, the methods in this chapter have also been validated using the results of [18]. It can be concluded that the finite element model used in this study accurately reflects the actual distribution of WRS in the component, enabling subsequent parameter analysis. It is noteworthy that the finite element model in this paper makes a certain assumption and simplification, such as assuming that the weld and base metal have the same conductivity and ignoring the effects of phase changes.

(7) $$\mathrm{MAE}=\frac{\sum_{i=1}^{n}\left|rs_{\mathrm{FEM}}-rs_{\mathrm{EXP}}\right|}{n}$$

In the equation, *rs*_FEM_ and *rs*_EXP_ represent the WRS results from FEM and EXP, respectively; and *n* represents the number of measured points.

#### 2.2.2. Key Parameter Analysis

**Figure 11:**
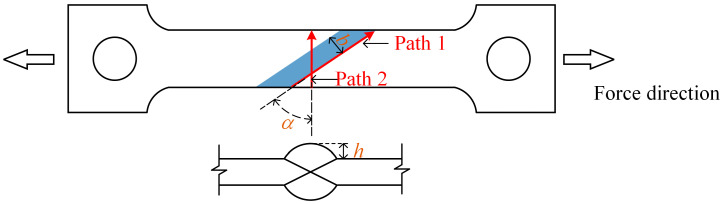
Parameter and path diagram.

In this section, based on the refined finite element model described above, the influence of parameters such as the inclined weld angle *α*, weld width *b*, and height *h* on the WRS distribution is systematically investigated. The relevant parameter definitions are shown in Figure 11. Since fatigue cracks in the inclined weld plate always initiate at the weld toe with the highest stress concentration and then propagate in a direction perpendicular to the applied load after a certain distance [27], two paths are defined: Path1 along the weld direction; and Path2 perpendicular to the applied load direction. Additionally, considering that crack propagation is mainly influenced by the WRS perpendicular to the crack plane [28], the normal WRS was defined, which referred to the WRS direction perpendicular to the path. The WRS is assumed to be uniform in the thickness direction. Therefore, this study focuses on the plane located at half the thickness for extracting the results.

A total of 25 models were studied, as shown in Table 1. The naming convention for the models is as follows: the digit following *α* represents the inclined weld angle, ranging from 0 to 60°; the digit following *b* represents the weld width; the digit following *h* represents the weld height; the ranges for *b* and *h* are based on the standard JB/T7949-1999 [29]; and *a*_f_, *a*_r_, *b*_h_, and *c* are shape parameters for the heat source. These parameters were adjusted to match the simulated weld pool shape with the modeled weld dimensions.

**Figure 12:**
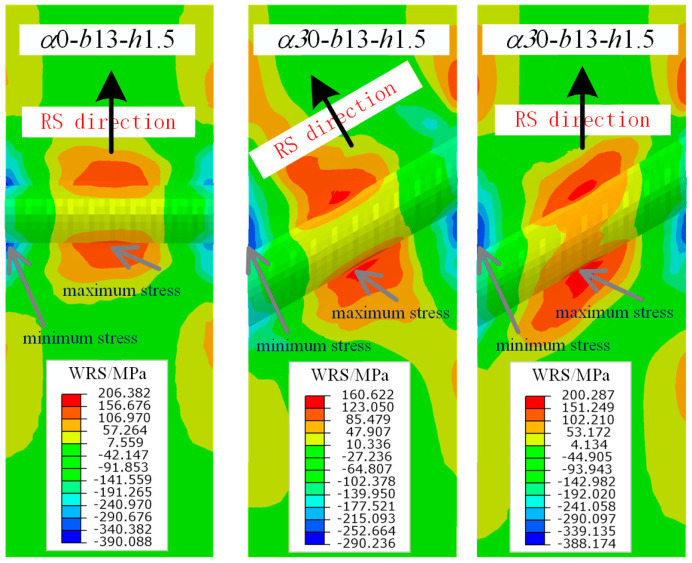
Stress contours of straight and inclined butt weld.

The WRS contour plots of the *α*0-*b*13-*h*1.5 and *α*30-*b*13-*h*1.5 models are selected as representative examples for straight weld and inclined weld comparison, respectively. For the inclined weld, the WRS in two directions is considered, as shown in Figure 12: perpendicular to the path direction and perpendicular to the assumed loading direction. It can be observed that the WRS distribution in the straight weld is axially symmetric, while the WRS distributions in both directions of the inclined weld are centrally symmetric. The maximum surface tensile WRS occurs at the middle of the weld toe, while the maximum surface compressive WRS appears at the end of the weld toe.

**Figure 13:**
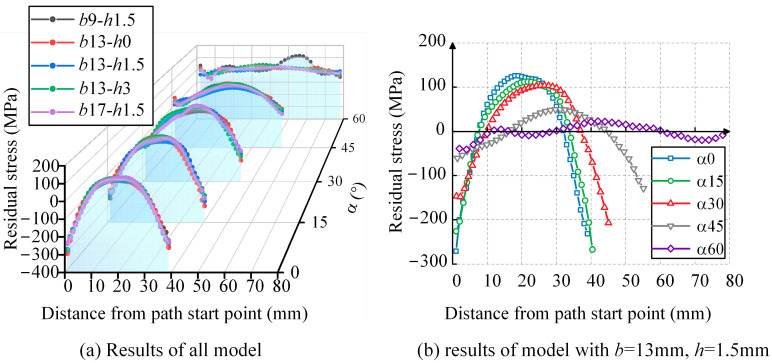
The WRS distribution perpendicular to Path1.

The normal WRS results along the Path1 direction are extracted. Figure 13a represents the calculated results for all models, where the α axis denotes the weld angle, and each angle corresponds to five curves in the cross-section, representing different combinations of weld width and height. To further illustrate the influence of the weld angle parameter, the *b*13-*h*1.5 curve for all angles is projected along the α axis in Figure 13b. The results are as follows:

(1) The WRS distribution in the inclined weld is symmetric to the perpendicular line on the path and exhibits a parabolic shape. The vertex of the parabola, located at the midpoint of the path, represents tensile stress, while the sides represent compressive stress.
(2) The weld angle significantly affects the magnitude of the WRS. As the angle increases, the tensile WRS at the midpoint of the path gradually decreases. In Figure 13b, the tensile WRS at the midpoint of the path for the inclined weld with angles of 0°, 15°, 30°, 45°, and 60° is 0.32*f*_y_, 0.29*f*_y_, 0.26*f*_y_, 0.13*f*_y_, and 0.06*f*_y_, respectively. When the angle changes from 0° to 30°, the tensile stress decreases from 0.32*f*_y_ to 0.26*f*_y_, and when the angle changes from 30° to 60°, the tensile stress decreases from 0.26*f*_y_ to 0.06*f*_y_. Overall, the magnitude of the tensile stress shows a decreasing trend with increasing angle, with a more significant change occurring in the latter range.
(3) The influence of weld height and width on the tensile stress is limited. For each angle in Figure 13a, the difference between the maximum and minimum stress values at the midpoint of the path, corresponding to the five curves, is 0.02*f*_y_, 0.08*f*_y_, 0.10*f*_y_, 0.09*f*_y_, and 0.09*f*_y_, respectively.

**Figure 14:**
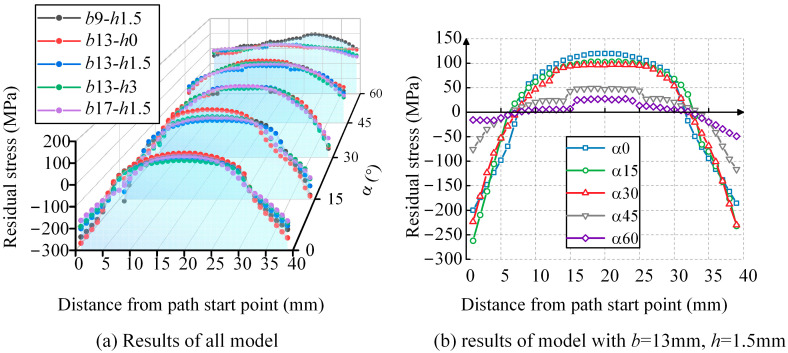
The WRS distribution perpendicular to Path2.

The normal WRS results along the Path2 direction are extracted. Figure 14a represents the calculated results for all models, where the α axis denotes the weld angle, and each angle corresponds to five curves in the cross-section, representing different combinations of weld width and height. To further illustrate the influence of the weld angle parameter, the *b*13-*h*1.5 curve for all angles is projected along the α axis in Figure 14b. The results indicate the following:

(1) The WRS distribution along this path also exhibits a parabolic shape that is symmetric with respect to the perpendicular line on the path. The vertex of the parabola, located at the midpoint of the path, represents tensile stress, while the ends represent compressive stress.
(2) Similar to the results along Path1, the weld angle significantly affects the magnitude of the WRS. As the angle increases, the tensile WRS at the midpoint of the path gradually decreases. In Figure 14b, the residual tensile stress at the midpoint of the path for the inclined weld with angles of 0°, 15°, 30°, 45°, and 60° is 0.31*f*_y_, 0.27*f*_y_, 0.25*f*_y_, 0.12*f*_y_, and 0.07*f*_y_, respectively. When the angle changes from 0° to 30°, the tensile stress decreases from 0.31*f*_y_ to 0.25*f*_y_, and when the angle changes from 30° to 60°, the tensile stress decreases from 0.25*f*_y_ to 0.07*f*_y_. Overall, the magnitude of the tensile stress shows a decreasing trend with increasing angle, with a more significant change occurring in the latter range.
(3) The influence of weld height and width on the tensile stress is relatively limited. For each angle in Figure 14a, the difference between the maximum and minimum stress values at the midpoint of the path, corresponding to the five curves, is 0.08*f*_y_, 0.14*f*_y_, 0.04*f*_y_, 0.09*f*_y_, and 0.09*f*_y_, respectively.
(4) The normal WRS distribution along Path1 and Path2 is similar, and it exhibits similar trends in response to the weld angle, width, and height.

#### 2.2.3. Influence Mechanism of Key Parameters

**Figure 15:**
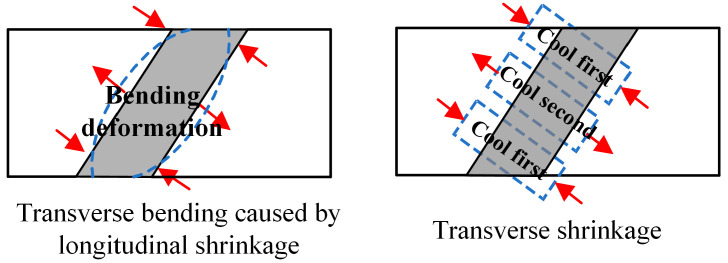
The formation mechanism of WRS.

According to the previous analysis, it can be concluded that the influence of the weld angle on WRS is mainly reflected in the peak values. To understand the mechanism behind the influence of the weld angle, this section first analyzes the formation mechanism of WRS. The mechanism behind the formation of WRS distribution perpendicular to the weld path is as follows: during the cooling stage, the steel plate undergoes longitudinal and transverse shrinkage. The longitudinal shrinkage results in transverse bending deformation of the two steel plates, as shown in Figure 15. The arrows represent the direction of force on the weld. This bending deformation is constrained by the weld, resulting in tensile stress in the middle of the weld and compressive stress at the ends. When the steel plate undergoes transverse shrinkage, the portions that cool first recover from the high-temperature plastic state to the low-temperature elastic state, hindering the shrinkage of the later-cooling portions. As a result, the later-cooling portions are under tension, while the earlier-cooling portions are under compression. In this model, the ends of the weld cool faster due to better heat dissipation conditions, resulting in compressive stress, while the middle portion experiences tensile stress. Therefore, the magnitude of WRS is closely related to the input of welding heat and cooling rate.

**Figure 16:**
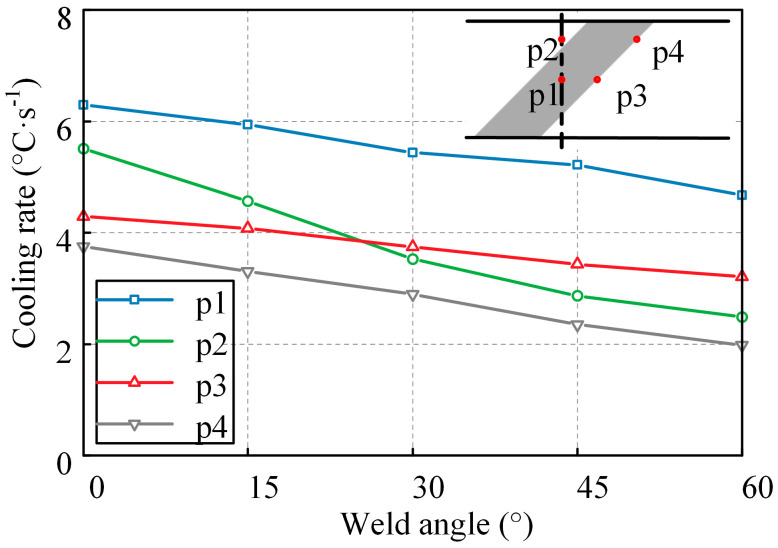
The relationship between cooling rate and weld angle at different points.

As the weld angle increases, the length of the weld increases, and the heat source stays on the weld for a longer time, resulting in a higher total heat input. Taking the *α*0-*b*13-*h*1.5, *α*15-*b*13-*h*1.5, *α*30-*b*13-*h*1.5, *α*45-*b*13-*h*1.5, and *α*60-*b*13-*h*1.5 models as examples, four typical points are selected from each model and named p1, p2, p3, and p4. The average cooling rates during the third pass welding stage from the highest temperature to 200 °C are extracted for these four points, as shown in Figure 16. It can be observed that as the angle α increases, the cooling rates at the middle and ends of the weld significantly decrease. This can be attributed to the decrease in peak WRS with increasing angle. At the same time, since the changes in weld height and width have a limited influence on the heat input, the variations in weld width and height have a limited impact on the resulting WRS when the weld angle remains unchanged.

## 3. Tension Behavior of CFRP-Strengthened Welded Plates

### 3.1. Model Details

**Figure 17:**
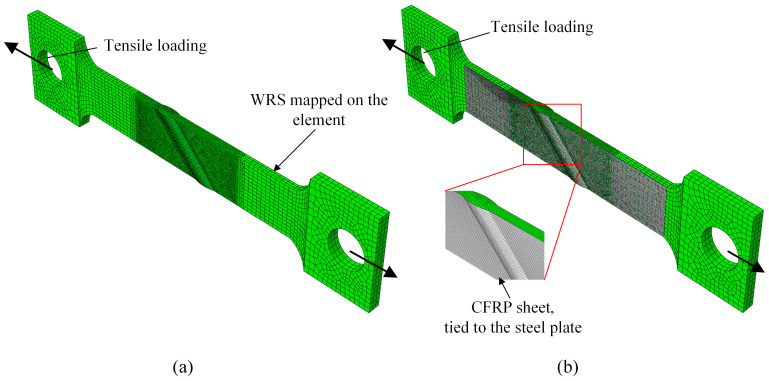
Tensile performance analysis FEA model for example of a45-b13-h1.5: (**a**) before CFRP strengthening; (**b**) after CFRP strengthening.

The *α*0-*b*13-*h*1.5, *α*15-*b*13-*h*1.5, *α*30-*b*13-*h*1.5, *α*45-*b*13-*h*1.5, and *α*60-*b*13-*h*1.5 models were selected for the tensile performance analysis. The finite element mesh size for the steel material is the same as that used for the WRS analysis model, facilitating the mapping of WRS onto the mesh. The CFRP properties are based on the HM-30 product from Shanghai Horse Construction Co., Ltd., Shanghai, China, with a double-sided single-layer thickness of 0.167 mm, elastic modulus of 240 GPa and diameter of 6 μm. The epoxy resin used is HM-180C3P, also from Shanghai Horse Construction Co., Ltd. CFRP strengthening was achieved by a vacuum curing process following reference [30], ensuring a strong bond between the CFRP and steel. Assuming that no failure or damage occurs at the interface between CFRP and the steel plate, the CFRP and steel plates are connected using tie constraints. The mesh size is set to 1 mm, as shown in Figure 17. Tensile force is applied to the end section of the steel plate, gradually increasing.

### 3.2. Results and Discussion

**Figure 18:**
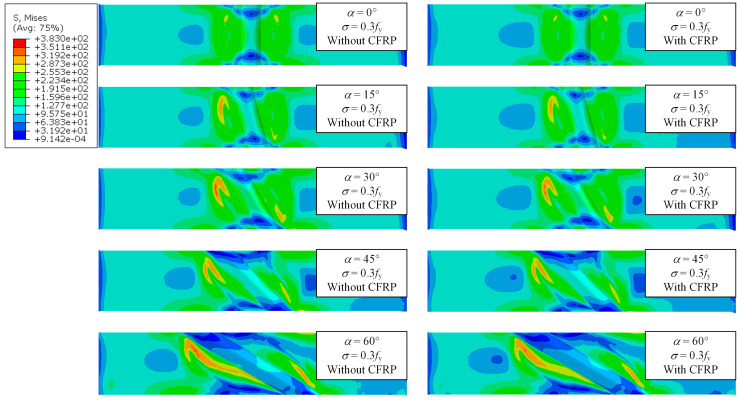
Stress contour of specimens under *σ* = 0.3*f*_y_.

**Figure 19:**
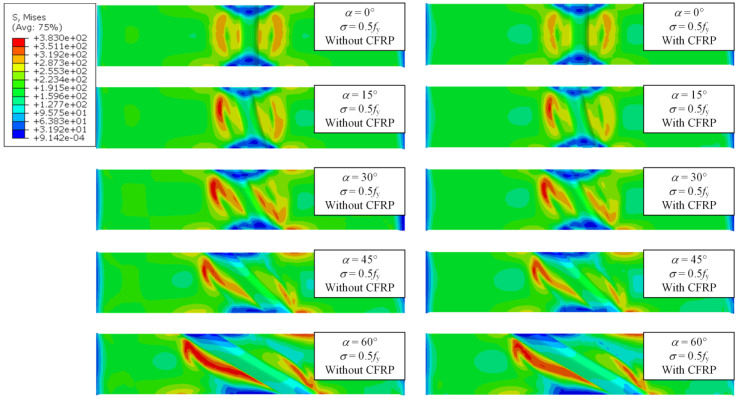
Stress contour of specimens under *σ* = 0.5*f*_y_.

**Figure 20:**
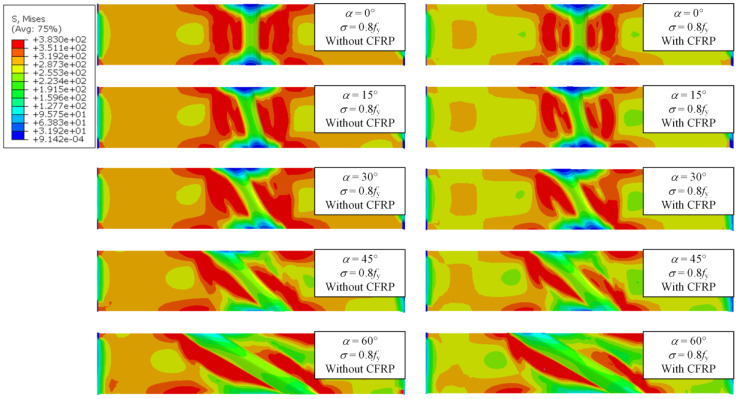
Stress contour of specimens under *σ* = 0.8*f*_y_.

Under the combined effect of WRS field and tensile stress field, the specimens are in a multiaxial stress state. Thus, the mises stress is used to describe the stress state of the specimen. Figure 18, Figure 19 and Figure 20 show the stress contour results of the specimens when the cross-sectional tensile stress *σ* = 0.3, 0.5, 0.8 times the yield strength, respectively. It can be observed that when *σ* = 0.3*f*_y_, the specimens are still mainly in the elastic stage, and the maximum stress appears near the weld. However, the stress is close to the yield stress due to WRS. When *σ* = 0.5*f*_y_, striped yielding zones appear near the weld. When *σ* = 0.8*f*_y_, the material continues to yield, and the yielding zone gradually extends towards the fixed end. The results indicate that the stress at the weld zone is relatively lower compared to the basemetal zone. This is because the cross-section at the weld is thicker, resulting in higher stiffness. In addition, the stress at both ends of the weld is close to zero. This is because the ends of the weld itself have significant compressive WRS, which counteracts the tensile stress from the applied load. Therefore, the ends of the weld enter the yielding state later than the middle part of the weld. As the weld angle increases and the length of the weld increases, the stress in the weld zone decreases, while the stress in the base material zone correspondingly increases. This is because a larger weld angle leads to a smaller WRS in the weld zone, which also means that the base material around the weld may absorb more of the stress as it supports the structure and accommodates the thermal expansion and contraction of the weld. CFRP strengthening reduces the stress of specimens, thereby delaying its entry into the yielding or failure state. The larger the stress, the more pronounced the reduction in effectiveness.

## 4. Conclusions

This study successfully measured the WRS in welded steel plates and developed a comprehensive FEM to simulate WRS in inclined weld plates. Subsequently, a simulation of the tensile behavior of a CFRP-strengthened model is achieved. Key findings are as follows:

(1) Experimental measurements revealed the WRS distribution. Along the path parallel to the weld, the transverse WRS distribution exhibits tensile stress in the middle and tends towards compressive stress at the ends; the longitudinal WRS is compressive in the middle and tensile at the ends. Along the path perpendicular to the weld, the transverse WRS distribution is characterized by tensile stress at the weld zone, gradually transitioning from tensile stress to compressive stress as the distance from the center of the weld increases.
(2) The developed FEM accurately simulated the welding process, aligning closely with experimental data. It was found that the weld angle significantly influences the peak tensile WRS, while the effect of weld width and height is minimal.
(3) The application of a double-sided single-layer CFRP sheet mainly affected the tensile stress levels without significantly altering the stress distribution pattern. The WRS was a key factor influencing its behavior, with the maximum stress consistently near the weld under tension. Stress variations were observed with changes in weld angle and length, affecting the stress distribution between the weld zone and the base material.

## Figures and Tables

**Table 1:** Parameter research models.

Model	*α*/°	*b*/mm	*h*/mm	*a_f_*/mm	*a_r_*/mm	*b_h_*/mm	*c*/mm
*α*0-*b*9-*h*1.5	0	9	1.5	4	8	4	5
*α*0-*b*13-*h*0	0	13	0	6	12	6	4
*α*0-*b*13-*h*1.5	0	13	1.5	6	12	6	5
*α*0-*b*13-*h*3	0	13	3	6	12	6	6
*α*0-*b*17-*h*1.5	0	17	1.5	7.5	15	7.5	5
*α*15-*b*9-*h*1.5	15	9	1.5	4	8	4	5
*α*15-*b*13-*h*0	15	13	0	6	12	6	4
*α*15-*b*13-*h*1.5	15	13	1.5	6	12	6	5
*α*15-*b*13-*h*3	15	13	3	6	12	6	6
*α*15-*b*17-*h*1.5	15	17	1.5	7.5	15	7.5	5
*α*30-*b*9-*h*1.5	30	9	1.5	4	8	4	5
*α*30-*b*13-*h*0	30	13	0	6	12	6	4
*α*30-*b*13-*h*1.5	30	13	1.5	6	12	6	5
*α*30-*b*13-*h*3	30	13	3	6	12	6	6
*α*30-*b*17-*h*1.5	30	17	1.5	7.5	15	7.5	5
*α*45-*b*9-*h*1.5	45	9	1.5	4	8	4	5
*α*45-*b*13-*h*0	45	13	0	6	12	6	4
*α*45-*b*13-*h*1.5	45	13	1.5	6	12	6	5
*α*45-*b*13-*h*3	45	13	3	6	12	6	6
*α*45-*b*17-*h*1.5	45	17	1.5	7.5	15	7.5	5
*α*60-*b*9-*h*1.5	60	9	1.5	4	8	4	5
*α*60-*b*13-*h*0	60	13	0	6	12	6	4
*α*60-*b*13-*h*1.5	60	13	1.5	6	12	6	5
*α*60-*b*13-*h*3	60	13	3	6	12	6	6
*α*60-*b*17-*h*1.5	60	17	1.5	7.5	15	7.5	5

## Data Availability

All data used to support the findings of this study are available from the corresponding author upon request.
